# Mucinous Cystic Neoplasm of the Pancreas in Pregnancy: A Case Report

**DOI:** 10.7759/cureus.50446

**Published:** 2023-12-13

**Authors:** Khalid Al Shamousi, Said A Al-Busafi, Masoud Kashoob, Adil Al Zadjali, Hoor Al Kaabi

**Affiliations:** 1 Medicine, Sultan Qaboos University, Muscat, OMN; 2 Gastroenterology, Sultan Qaboos University Hospital, Muscat, OMN; 3 Medicine, Oman Medical Specialty Board, Muscat, OMN; 4 General Surgery, Sultan Qaboos University, Muscat, OMN

**Keywords:** case report, acute pancreatitis, pregnancy, pancreas, mucinous cystic neoplasm

## Abstract

Mucinous cystic neoplasms (MCNs) of the pancreas are rare epithelial neoplasms, characterized by an inner epithelial layer and an ovarian-type sub-epithelial stroma. These lesions are typically benign but can pose challenges during pregnancy due to their rapid growth potential, associated risk of malignant transformation, and complications such as pancreatitis. We present a case of a 39-year-old pregnant female with a history of recurrent acute pancreatitis, diagnosed with an MCN during pregnancy. Diagnostic procedures were deferred until after delivery, followed by successful distal pancreatectomy. This case underscores the importance of individualized management strategies in pregnant patients with pancreatic MCNs, balancing the need for timely intervention with maternal and fetal safety. Long-term follow-up is generally unnecessary for MCNs without associated invasive carcinoma, emphasizing the favorable prognosis of these lesions following complete surgical resection.

## Introduction

Mucinous cystic neoplasms (MCNs) of the pancreas, known for producing mucin, are epithelial neoplasms that can precede invasive pancreatic cancer. Typically, these neoplasms do not connect with the pancreatic ductal system [1,2]. MCNs are characterized by two distinctive histological components: an internal epithelial layer and a unique ovarian-type sub-epithelial stroma, composed of spindle-shaped cells with varying nuclei shapes and sparse cytoplasm [1,2]. This ovarian-type stroma is not only a defining feature of MCNs but also a crucial diagnostic criterion.

Although relatively rare, MCNs constitute about 8% of all surgically removed cystic neoplasms of the pancreas, predominantly found in females aged 40 to 60 years [1,2].

Notably, MCNs are frequently encountered in pregnant patients, presenting challenges in diagnosis, management, and the timing of surgical intervention [3]. In this context, we report a case of a pregnant patient with an MCN, initially managed conservatively and subsequently undergoing successful post-delivery surgery.

## Case presentation

In January 2021, a 39-year-old female, 30 weeks into her pregnancy, presented at the emergency department of Sultan Qaboos University Hospital-Oman with a two-day history of epigastric pain radiating to her back, accompanied by nausea and vomiting.

Her medical history included gestational diabetes and multiple episodes of acute pancreatitis, first diagnosed in 2015. During the initial episode, she presented at the Army Forced Hospital-Oman with similar symptoms and elevated lipase levels. An abdominal ultrasound at that time revealed no significant findings. However, a subsequent CT scan of the abdomen identified a cyst in the pancreatic tail, leading to a referral to Royal Hospital for further evaluation using endoscopic ultrasound (EUS).

In December 2020, EUS at Royal Hospital-Oman revealed a unilocular, multiloculated lesion at the junction of the pancreas's body and tail, measuring 39 x 35 mm. This lesion, characterized by internal thin septations and devoid of any solid mass, raised differential diagnoses including mucinous cyst adenoma and a macrocystic variant of serous cyst adenoma. A follow-up EUS and aspiration were planned in three months but were deferred due to the patient's pregnancy, with a reschedule planned post-delivery.

Upon assessment in our ED in January 2021, her primary complaint was worsening epigastric pain postprandially. Physical examination revealed moderate abdominal pain, with vital signs as follows: Temperature 36.4°C, blood pressure 120/58 mmHg, heart rate 75 bpm, respiratory rate 20 bpm, and oxygen saturation 99% in room air. The abdomen was soft with tenderness in the epigastric area, while other systematic examinations were unremarkable.

The laboratory results shown in Table 1 indicated a hemoglobin level of 10.5 g/dL, slightly increased neutrophils at 6.3 × 10^9/L with a normal white blood cell count, and elevated lipase at 572 U/L. Liver function tests were within normal limits. An abdominal ultrasound revealed a well-defined hypoechoic cystic lesion, approximately 7 cm x 6.3 cm in size, adjacent to the pancreatic tail with multiple internal thin septa and no significant solid components or vascularity on Doppler ultrasound. There was no evident pancreatic duct dilatation.

**Table 1:** Summary of the laboratory test results upon first admission Hb, hemoglobin; APTT, activated partial thromboplastin time; PT, prothrombin time; ALT, alanine aminotransferase; AST, aspartate aminotransferase; ALP, alkaline phosphatase; GGT, gamma-glutamyl transferase.

Test	Result	Normal range
Hb (g/L)	10.5	11-14.5
Haematocrit (L/L)	0.335	0.34-0.43
Platelet count (10^9^ /L)	341	150-450
White cell count (10^9^ /L)	7.3	2.4-9.5
Neutrophils 10^9/L	6.3	1-4.8
PT (sec)	10.5	9.8-12
APTT (sec)	31.1	25-36.4
ALT (U/L)	17	0-33
AST (U/L)	18	0-32
ALP (U/L)	47	35-104
GGT (U/L)	3	6-42
Bilirubin (umol/L)	3	0-17
Lipase (U/L)	572	13-60

The patient was admitted under the impression of acute pancreatitis and managed conservatively with hydration and intravenous analgesia, showing improvement within two days. She was subsequently discharged with a follow-up plan for the pancreatic cyst at Royal Hospital.

During July and August 2021, she experienced two more episodes of pancreatitis during her pregnancy, treated conservatively in our ED. Post-delivery, the planned MR pancreas and EUS +- aspiration were conducted.

**Figure 1:**
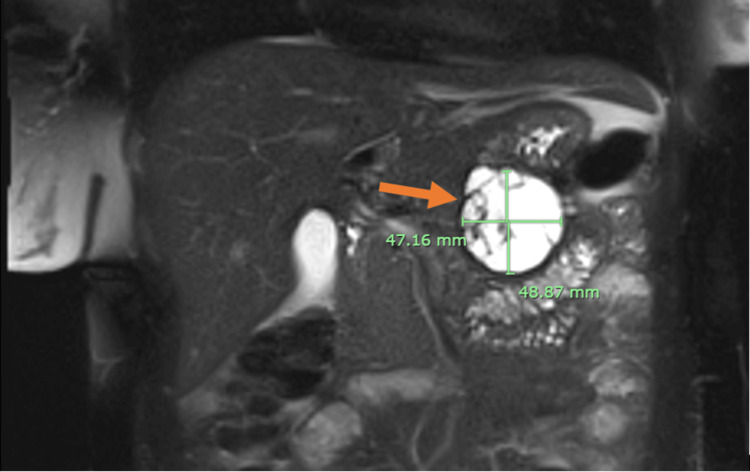
MRI image Arrow indicating a well-defined, rounded multicystic lesion in the pancreatic tail, measuring approximately 48 x 49 mm

The patient had an uncomplicated delivery on September 9, 2021. On September 17, 2021, she returned to the ED with similar symptoms and was admitted for acute pancreatitis. MRI of the pancreas (Figure 1) revealed a well-defined abnormal signal intensity rounded cystic lesion in the pancreatic tail, measuring approximately 48 mm x 49 mm. An EUS identified a 57 mm x 49 mm round macrocystic anechoic cyst in the distal body/tail of the pancreas with multiple thick septations and a suspected mural nodule. Fine Needle Aspiration (FNA) biopsy of the nodule was performed. The cystic fluid analysis (Table 2) showed a significantly elevated amylase level of 19,445 U/L and a high carcinoembryonic antigen (CEA) level of 8,284.83 ug/L, with the fluid culture showing no growth and the presence of mucous and inflammatory cells, but no epithelial cells. Considering the diagnostic results, a diagnosis of an MCN was established, necessitating a distal pancreatectomy as the appropriate surgical intervention.

**Table 2:** Laboratory findings for the pancreatic cyst fluid analysis

Test	Result	Unit
Fluid Amylase	19,445	U/L
Fluid Carcinoembryonic Antigen (CEA)	8,284.83	ug/L
Fluid Culture	No growth	-
Pancreatic Cyst Fluid Analysis	Mucous and inflammatory cells, no malignant cells	-

In October 2021, the patient underwent a successful distal pancreatectomy. Post-operatively, she reported significant symptom improvement and required no further hospital admissions. Histopathology revealed mucinous and inflammatory cells. A follow-up MRI in December 2022 confirmed no evidence of local residual or recurrent disease.

## Discussion

Pancreatic MCNs are relatively rare, typically benign, and exhibit a slow growth rate, generally not infiltrating surrounding tissues. Most MCNs are found in women, with a female-to-male occurrence ratio of 20:1, and they are typically diagnosed at an average age ranging between 40 and 50 years [1]. Their occurrence during pregnancy, though infrequent, has been documented in a limited number of case reports [1]. Notably, several studies have observed a rapid increase in the growth rate of MCNs during pregnancy [4-7].

The ovarian-type stroma of pancreatic MCNs, characterized by the expression of estrogen receptors and progesterone receptors [5-7], suggests that hormonal influence during gestation may contribute to this accelerated growth. In the case presented, the MCN size increased significantly from 3.9 x 3.5 cm pre-pregnancy to 7.0 x 6.3 cm in the third trimester.

This rapid growth raises concerns about the potential for malignant transformation into invasive carcinomas, increased risk of pancreatitis, and other complications such as tumor rupture or fetal hazards like intrauterine growth restriction [6,7]. Although the direct impact of elevated pregnancy hormones on the malignant transformation of MCNs is not fully understood, Farahmandi et al. reported a higher incidence of invasive carcinoma within MCNs during pregnancy compared to non-pregnant cases [4]. Additionally, progesterone has been suggested to play a protective role by potentially suppressing malignant transformation in the ovarian-like stroma of MCNs [8-10].

Diagnostically, mucinous neoplasms present a challenge due to their internal heterogeneity [11]. This necessitates a cautious approach in both diagnosis and treatment to safeguard the health of both mother and fetus. In the case presented, significant diagnostic procedures, such as EUS and decisions regarding surgical intervention, were postponed until after delivery, aligning with the 2018 European evidence-based guidelines [12]. This approach ensured that no alarming features were overlooked while prioritizing patient and fetal safety.

Post-delivery, a comprehensive evaluation was undertaken to inform the surgical decision-making process. The outcomes of these diagnostic procedures, their findings, and their specific influence on opting for distal pancreatectomy would provide valuable insights for similar cases in the future.

In terms of prognosis, MCN patients typically have favorable outcomes post-operatively. The five-year survival rate post-complete surgical resection of MCNs, in the absence of associated invasive carcinoma, is nearly 100%. This exceptional prognosis, coupled with a negligible risk of tumor recurrence, suggests a less intensive follow-up regimen may be adequate [13].

## Conclusions

In conclusion, this case report highlights the rare occurrence of an MCN of the pancreas in a pregnant patient, presenting unique challenges in diagnosis and management. MCNs are typically benign and slow-growing, but during pregnancy, their rapid expansion is likely influenced by hormonal changes. While the direct correlation between female sex hormones and MCN transformation remains unclear, the risk of malignant transformation during pregnancy underscores the importance of vigilant monitoring and timely intervention.

Our patient's case demonstrates the complexities involved in managing pancreatic cystic neoplasms during pregnancy. Delaying diagnostic procedures until after delivery, when clinically appropriate, can minimize risks to both the mother and the fetus. Surgical excision, in accordance with established guidelines, remains a crucial intervention for MCNs associated with invasive carcinoma.

Timely diagnosis, careful monitoring, and individualized management strategies are essential in ensuring the best outcomes for pregnant patients with pancreatic MCNs. Long-term follow-up post-surgery is often unnecessary for MCNs without associated invasive carcinoma, as these lesions typically have excellent prognoses.

## References

[1] Nilsson LN, Keane MG, Shamali A (2016). Nature and management of pancreatic mucinous cystic neoplasm (MCN): a systematic review of the literature. Pancreatology.

[2] (2010). WHO Classification of Tumours of the Digestive System.

[3] Revoredo F, de Vinatea J, Reaño G, Villanueva L, Kometter F, Arenas J, Polanco PM (2020). Mucinous cystic neoplasms of the pancreas associated with pregnancy: two case reports. Medicine (Baltimore).

[4] Farahmandi S, Elessawy M, Bauerschlag DO (2021). Mucinous cystic neoplasm of pancreas in a pregnant woman presenting with severe anemia and gastric bleeding: case report and review of the literature. Healthcare (Basel).

[5] Esposito I, Schlitter AM, Sipos B, Klöppel G (2015). Classification and malignant potential of pancreatic cystic tumors (Article in German). Pathologe.

[6] Kato M, Kubota K, Kita J (2005). Huge mucinous cystadenoma of the pancreas developing during pregnancy: a case report. Pancreas.

[7] Naganuma S, Honda K, Noriki S (2011). Ruptured mucinous cystic neoplasm with an associated invasive carcinoma of pancreatic head in a pregnant woman: report of a case and review of literature. Pathol Int.

[8] López-Tomassetti Fernández EM, Martín Malagón A, Arteaga Gonzalez I, Muñiz Montes JR, Díaz Luis H, González Hermoso F, Carrillo Pallares A (2005). Mucinous cystic neoplasm of the pancreas during pregnancy: the importance of proper management. J Hepatobiliary Pancreat Surg.

[9] Thompson LD, Becker RC, Przygodzki RM, Adair CF, Heffess CS (1999). Mucinous cystic neoplasm (mucinous cystadenocarcinoma of low-grade malignant potential) of the pancreas: a clinicopathologic study of 130 cases. Am J Surg Pathol.

[10] Robles-Diaz G, Duarte-Rojo A (2001). Pancreas: a sex steroid-dependent tissue. ISR Med Assoc J.

[11] Brown TH, Menon VS, Richards DG, Griffiths AP (2009). Gastrointestinal bleeding in a pregnant woman: mucinous cystic neoplasm of pancreas mimicking gastrointestinal stromal tumor of stomach. J Hepatobiliary Pancreat Surg.

[12] (2018). European evidence-based guidelines on pancreatic cystic neoplasms. Gut.

[13] Testini M, Gurrado A, Lissidini G, Venezia P, Greco L, Piccinni G (2010). Management of mucinous cystic neoplasms of the pancreas. World J Gastroenterol.

